# Effects of yoga and add on Ayurvedic *Kati Basti* therapy for patients with chronic low back pain: A randomized controlled trial

**DOI:** 10.1016/j.jaim.2024.101030

**Published:** 2024-08-24

**Authors:** Maheshwor Bhatta, Suchitra S. Patil, Sunil Singh Yadav, Sangeeth Somanadhapai, Rita Thapa

**Affiliations:** a Swami Vivekananda Yoga Anusandhana Samsthana, Bengaluru, India; b Department of Yoga and Life Science, Swami Vivekananda Yoga Anusandhana Samsthana, Bengaluru, India; c Faculty of Naturopathy and Yogic Sciences, SGT University, Gurgaon, India

**Keywords:** CLBP, Ayurveda therapy, Kati basti, Ksheerbala taila, Yoga therapy

## Abstract

**Background:**

Chronic low back pain (CLBP) signficantly affects quality of life and productivity, leading to limitations in mobility, activity, and potential work absenteesim. Yoga and Ayurveda have shown promising evidence in reducing pain, improve function, and enhancing well-being for individuals with CLBP, as demonstrated by numerous studies.

**Objectives:**

The aim of this study is to assess the effect of a 1-week residential integrative approach to yoga therapy. (IAYT) alone versus when combined with Ayurveda therapy (*Kati Basti*) in patients with CLBP.

**Methods:**

Forty patients were recruited from E-section of a holistic health center in South India for randomization and split into a Yoga and Ayurveda (*n*=20) and a Yoga-only (*n*=20) group. Yoga and Ayurveda group received a 1-week residential program combining Yoga and Ayurveda (therapy including *Kati Basti with Ksheerbala Taila*), while the Yoga-only group received only yoga therapy. Assessments at baseline, 1-week, and three months were measure pain intensity, disability, and depression.

**Results:**

Both Yoga and Ayurveda group, and Yoga-only group showed significant reductions in pain and improvements in disability and depression at 1-week and three months. Quality of life also improved, specifically in physical, social, environmental, and psychological health. No significant differences were found between the groups in terms of pain, disability, and depression.

**Conclusion:**

Both interventions demonstrated comparable results in pain reduction and disability improvement, as well as alleviating depression symptoms. Only Yoga and Ayurveda group exhibited improvement in physical health. Further research should explore long-term effects and compare different yoga interventions.

## 1 Introduction

Low back pain (LBP) is a common symptom not associated with any particular illness. It can be recognized by the location of the pain, usually between the creases of the buttocks and the lower ribs [1]. Many underlying disorders that affect anatomical structures such muscles, nerve roots, bones, joints, intervertebral discs (IVDs), and abdominal organs are frequently the cause of it [2].

While certain causes of back pain, including radiculopathy or spinal stenosis, can be linked to the condition, most occurrences of low back pain are considered non-specific since their etiology is unknown [3]. LBP recurrence rates in the working population vary from 20% to 44% in a year to as high as 85% throughout a lifetime.

A case of chronic low back pain (CLBP) is any LBP that persists for more than three months or beyond the average recovery period [4]. It is a serious issue that is spreading around the globally and worsening, partly because of the aging and expanding global population. It is a condition that affects individuals of all ages and is commonly associated with smoking, obesity, sedentary jobs, and low socioeconomic status [5]. At some point in their life, between 70% and 80% of adults will experience CLBP [6]. Women have a roughly 50% greater prevalence of CLBP compared to men [7]. Research indicates that 23% of adults globally experience persistent low back discomfort, and this cohort has shown a 24%–80% recurence rate. CLBP requires a multidisciplinary approach to diagnosis and treatment. The biopsychosocial method is the gold standard for treating non-specific CLBP [8].

Disability affects physical performance and, as a result, job productivity, making it a fundamental issue in CLBP [9]. It is quite difficult for people who have chronic pain to be physically active. Furthermore, chronic pain and psychological disorders often co-occur. When these illnesses are not properly treated, people often experience severe disabilities and a reduced quality of life [10]. Chronic pain negatively affects mental health and lowers quality of life overall; the most common co-occurring illnesses are depression and anxiety. Clinical depression is compounded when anxiety, pain, and depression are present.

Studies in the scientific community demonstrate that yogic therapies can effectively improve spinal mobility and decrease pain, analgesic use, and disability [11]. Yoga has been shown in numerous studies to be a suitable and safe intervention for musculoskeletal problems, with lower pain levels and improved functional outcomes [12]. The yogic lifestyle modification program is a very efficient technique to alleviate chronic low back pain compared to physical exercise [11]. When it comes to therapeutic effectiveness, yoga works better than physical therapy [13]. By improving spinal flexibility and strength, yoga can help individuals with persistent low back pain (LBP) [14]. It can also enhance patients' mental functioning, lessen pain catastrophizing, increase pain acceptance, and increase joint and muscular flexibility.

A recent study on the effects of yoga intervention showed lower blood pressure and an improvement in HRV [15]. An additional systematic review article incorporating Ayurveda and Yoga revealed noteworthy enhancements in quality of life, muscle strength, range of motion, and a decrease in pain, tension, and depressive symptoms [16].

Ayurveda, the oldest Eastern medical system, defines low back pain as the most common symptom of musculoskeletal disorders, called *Kati Soola* [17]. The musculoskeletal system is significantly impacted by this illness because Vata Dosha is activated. The Shleshmadhara Kala (joint betwwen the vertebrae) which is responsible to lubricate and reduce friction during movements of teh vertebral column by secreting Shesmaka Khapa [18]. It is explained that Kati Soola is a symptom of several musculoskeletal illnesses, including Vataja Shoola, Trika Vedana, Prushta Shools, Kat Vayu, Trika Graha, and Grudrasi Vata [17]. One of the common ayurvedic treatment for musculoskeletal disorders is *Kati Basti,* a kind of Snigdha Sveda, synchronized Snehana and Svedana. Svada causes perspiration to increase ansd also brings out Mala Dravy [19]. As a curative measure for the lumbar spine lesion, Snehana offers Snigdhata and Brimhana [20]. In *Kati Basti*, compression, irritation, or inflammation due to intervertebral disc degeneration and decreased Shesaka Kapha lubricating function result in discomfort. The primary cause of Vata Prakopa is degeneration of Dhatu Kshya. Thus, to balance the Vata Dosha, local snehana and svedana in the form of *Kati Basti* work wonders [21].

*Ksheerabala Taila* combined with *Kati Basti* is a well-liked Ayurvedic treatment for low back pain. Ksheerabala Taila is a significant Ayurvedic oil formulation that efficiently heals neurological diseases like hemiplegia, poliomyelitis, facial paralysis, and sciatica. It is prepared from cow milk, sesame oil, and bala (Sida cordifolia). The comparable drug-based formulation was first referenced in ancient writings Sahasrayogam. Charaka and Ashtanga Hridaya both referred to it as Shatasahasra Pakabala Taila and Shata pakabala-sahasrapakabala Taila, respectively [22].

Research backs up the beneficial effects of Ayurvedic treatment, *Kati Basti* and Yoga for CLBP. When combined, the two therapies may yield even better outcomes as they both seem beneficial in managing the illness alone. The premise that their synergistic actions increase therapeutic effectiveness underlies the claim of their combined efficacy. The purpose of this research is to evaluate the impact of 1-week of residential integrative approach to yoga therapy (IAYT) with or without combination of Ayurvedic therapy, *Katii Basti* on pain, disability, quality of life, and depression in individuals with persistent lower back pain.

## 2 Materials and methods

### 2.1 Trial design

The study was randomized using a computer-generated allocation series. Patients were split into two groups, one for Yoga and Ayurveda and other for Yoga-only, with a 1:1 allocation ratio.

### 2.2 Patients

Based on established qualifying criteria, patients were recruited for the study at S-VYASA University (Arogyadhama) in E-section, a holistic health center in South India. Twenty patients were chosen for the Yoga and Ayurveda group after a doctor's consultation. They were prescribed an IAYT and *Kati Basti* treatments with *Ksheerabala Taila*. Those in Yoga-only group were solely administered IAYT therapy.

The inclusion criteria for this study were both male and female patients between the ages of 18 and 65 who had been experiencing chronic back pain that had been pre-diagnosed for longer than three months, regardless of whether the pain radiating down the leg.

**Fig. 1:**
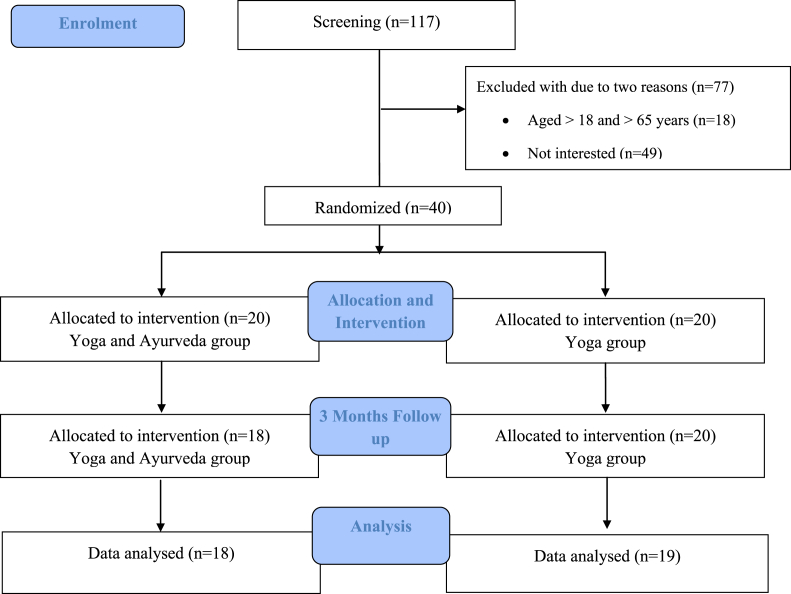
The trial profile of participants of this study.

Patients diagnosed with CLBP as a result of organic spine diseases, such as primary or secondary cancer, chronic infections as identified by lumbar spine X-rays, chronic diseases like tuberculosis, renal disease, hemorrhagic sickness, and acute rheumatoid arthritis were not included in this study. In the previous iteration of the study, patients who refused to enroll or who failed to meet the inclusion criteria were excluded. Fig. 1 (trial profile).

### 2.3 Sample size calculation

The sample size was estimated using the effect size of the Oswestry Low-Back Pain Disability Index (ODI), with post-mean and standard deviation values for the yoga group (18.70 ± 11.55) and the control group (35.75 ± 15.19). This calculation, based on a previous study, was conducted using G* power software, version 3.1.9.2, with a sample size of 102 patients, an alpha level of 0.05, and a power of 0.80 [23]. However, due to the limited number of patients, there were only forty patients in total, with 20 in the Yoga and Ayurveda group and 20 in the Yoga-only group.

### 2.4 Ethical considerations & trial registration

The institutional ethical committee granted ethical approval before the commencement of the study. The clinical trial was registered with the Clinical Trials Registry of India under registration numbers CTRI/2022/08/044600. Patients provided informed consent after receiving comprehensive information about the study's purpose, procedures, interventions, and rights, both verbally and in writing, in their preferred language.

### 2.5 Intervention

#### 2.5.1 Group 1: Yoga and Ayurveda

The 1-week residential yoga therapy (IAYT) and additional *Kati Basti* therapy were given to the Yoga and Ayurveda group.

##### 2.5.1.1 Integrative approach of yoga therapy (IAYT)

IAYT:, a 1-week intensive residential yoga program, included breathing exercises, meditation, asanas (physical postures) tailored to back pain, didactic, and interactive sessions covering the philosophical underpinnings of yoga. Yoga techniques were based on verified yoga modules [24], which are mentioned in Table 1 below.

**Table 1:** IAYT Module for chronic low back pain.

Sr.	Name of asana	Alternate name
1	*Supta udarakarsanasana*	Folded leg lumbar stretch
2	*Sava udarakarsanasana*	Crossed leg lumbar stretch
3	*Pavanamuktasana*	Wind releasing pose
4	*Setu bandhasana breathing*	Bridge pose lumbar stretch
5	Instant relaxation technique	Instant relaxation technique
6	*Vyaghrasana*	Tiger breathing
7	*Bhujangasana*	Serpent pose
8	*Salabhasana* breathing	Locust pose
9	*Uttanapadasana*	Straight leg raise pose
10	Quick relaxation techniques	Quick relaxation techniques
11	*Ardha-katicakrasana*	Lateral arc pose
12	*Ardha chakrasana*	Half wheel pose
13	Deep relaxation technique	Deep relaxation technique
14	*Nadi suddhi pranayama*	Alternate nostril breathing
15	*Bhramari pranayama*	Humming bee breath
16	*Vibhagiya pranayama*	sectional breathing
17	*Nadanusandhana*	A, U, M, AUM chanting
18	Om Meditation	Om Meditation
19	*Laghu Shankha Prakshalana* (yogic colon cleansing)-	On 3rd and 5th days of intervention.

For 1 week, the patients in the residential yoga program (IAYT) adhered to the daily schedule shown in Table 2.

**Table: 2:** Integrative approach of yoga therapy daily schedule.

Timing	Activity
05:30 a.m. to 06:00 a.m.	Om meditation
06:00 a.m. to 07:00 a.m.	Special technique (Yoga Module)
07:00 a.m. to 08:00 a.m.	Breakfast
08:00 a.m. to 09:50 a.m.	Lecture
10:00 a.m. to 11:00 a.m.	Parameters
11:00 a.m. to 11:50 a.m.	Pranayama
12:00 p.m. to 01:00 p.m.	Special technique (Yoga Module)/Yoga and Ayurveda group on Kati Basti
01:00 p.m. to 02:00 p.m.	Lunch
02:00 p.m. to 03:00 p.m.	Video/Counseling
03:00 p.m. to 03:50 p.m.	Cyclic meditation
04:00 p.m. to 05:00 p.m.	Special technique (Yoga Module)
05:15 p.m. to 06:00 p.m.	Tuning with nature
06:00 p.m. to 06:25 p.m.	Devotional session
06:30 p.m. to 07:30 p.m.	Tratak and MSRT
07:30 p.m. to 08:20 p.m.	Dinner
08:15 p.m. to 09:00 p.m.	Happy assembly (Saturday, Monday and Wednesday)

###### 2.5.1.1.1 Kati Basti therapy

For a week, *Kati Basti* therapy used *Ksheerbala* *Taila* prepared from internal Pharmacy of Sushrutha Ayurveda Medical College and Hospital, South India.

• *Require Materials: 1)* Masha powder (Black gram powder) – ½ kg to 1 kg. 2) Oil (*Ksheerbala Taila*) – 200 ml–500 ml. 3) Plastic or Steel ring – (2″ height and 18–20″ circumference)- *Kati Basti* rings of different sizes)

###### 2.5.1.1.2 Procedure of Kati Basti

• Purva Karma (Pre-operative Procedure): The patient should be asked to eliminate their urine and feces before being brought to the table.
• Pradhana Karma (Procedural Method): *Kati Basti* treatment involves cleaning the lumbar area while the patient is instructed to lie prone on a table. Black gram powder and water are combined to make a thick dough, which is then shaped into a slab-like form and secured with a steel or plastic ring to serve as a reservoir. The oil is gradually poured into the ring after being heated over a water bath to maintain a steady temperature. For 30 to 45 minutes, the patient lies with the dough structure filled with oil in place. Following that, 10 minutes of Snehana (oil application) and Nadi Sweda (steam therapy) are administered to the lumbosacral area.
• Paschat Karma (Post-Operative Procedure): After receiving *Kati Basti* therapy and subsequent removal of dough and oil, a gentle Mrudu Samvahana (oil massage) is administered without applying pressure to lumbar region, allowing the patient to undergo a period of rest.

#### 2.5.2 Group 2: yoga-only

The yoga group did not receive any further therapies or treatments other one the 1-week residential IAYT yoga program. Table 1 above mentions the same IAYT model with time seclusion.

### 2.6 Data extraction

In 2022, data was collected from August to November. On the day of the patients admittance, pre-data was collected following the completion of the eligibility criteria screening and the doctor consultation. Post data was collected on the 7th day of the therapy after the patient's discharge. Follow-up data was collected three months after the intervention all of the information was recieved using a Google form.

#### 2.6.1 Visual analog scale (VAS)

The visual analog scale (VAS) was employed as the instrument to gauge the patients' level of discomfort during the investigation. This scale ranged from 0 to 10, with 0 representing no pain, 2–3 denoting mild pain, 4–5 denoting moderate pain, 6–7 denoting strong pain, and 8–10 denoting excruciating agony.

#### 2.6.2 Oswestry Low-Back Pain Disability Index (ODI)

The questionnaire for the Oswestry Low-Back Pain Disability Index (ODI) was manually assessed by allocating values to each part and adding them up to get the total score. The final score was expressed as a percentage of disability by dividing the total score by the maximum possible score and multiplying the result by 100, yielding a scale range of 0–100%. Based on the persentage range, the handicap was categorized as minimal, moderate,severe, crippled, or bed-ridden/exaggerated [25].

#### 2.6.3 WHOQOL-BREF

With 26 measures, the WHOQOL-BREF is a comprehensive, standardized tool for evaluating quality of life. The scale measures a person's quality of life across four domains: environmental health (eight items), social relationships (three items), psychological health (six items), and physical health (seven items). Two questions are also included for the “general health” and “overall QOL” components. Higher values signify improved quality of life, and the domain scores are positively scaled. The range of scores for each domain is 4–20. The internal consistency of WHOQOL-BREF varied between 0.66 and 0.87 (Cronbach's alpha coefficient). It was found that the scale had good discriminant validity. Due to its high test-retest reliability, it is recommended for use in health surveys and for determining the optimal intervals at which to assess the effectiveness of any intervention.

#### 2.6.4 The Beck's Depression Inventory (BDI)

The 21-item Beck Depression Inventory (BDI) was manually scored using the statements as a basis. The sum of the individual item scores was then used to determine the final score, which ranged from 0 to 63. Based on predetermined ranges, the total score was defined as minimal depression, mild depression, moderate depression, severe depression [26].

### 2.7 Data analysis

Version 20 of the Statistical Package for Social Science (SPSS) was used for the statistical analysis. The Shapiro-Wilk test was used to determine whether the data were normally distributed, and the results confirmed that the data followd a normal distribution. Repeated measures ANOVA was carried out to evaluate comparisons between gruops, allowing for the examination of gruop differences over various time periods. Paired sample t-tests were also performed to evaluate within-gruop variations, specifically examining changes between the baseline and three months following the intervention, as well as between the baseline and follow-up.

## 3 Results

Variables were collected for the current study at baseline, following a week intervention of IAYT with add-on *Kati Basti* or IAYT alone, and at the three-month mark. The demographic differences between the two groups are displayed in Table 3. During the intervention, there were 20 patients in the Yoga and Ayurvedic group (12 males and 8 females) and 20 patients in the Yoga-only (10 males and 10 females). During the follow-up data collection, we were unable to contact three patients, one male and one female patient from Ayurvedic and Yoga group, and one female patient from the Yoga-only gruop. There was no discernible in age and gender between the two groups at baseline.

**Table 3:** Demographic characteristic of patients.

Characteristic	Yoga and Ayurveda Group	Yoga-only Group	Total
**Number of patients**	During Intervention = 20	During Intervention = 20	Intervention = 40
	Follow up = 18	Follow up = 19	Follow up = 37
**Genders**	Male = 12	Male = 10	Male = 22
	Female = 8	Female = 10	Female = 18
**Age (Mean** ± **SD)**	52.2 ± 10.71	52.1 ± 11.65	52.3 ± 11.05
**Education**	Primary = 4	Primary = 1	Primary = 5
	Higher Secondary = 3	Higher Secondary = 1	Higher Secondary = 4
	Graduate = 8	Graduate = 9	Graduate = 17
	Post Graduate = 5	Post Graduate = 9	Post Graduate = 14
**Work**	Sedentary = 13	Sedentary = 16	Sedentary = 29
	Non-Sedentary = 7	Non-Sedentary = 4	Non-Sedentary = 11

### 3.1 Yoga and Ayurveda

After one week and three months, the Yoga and Ayurveda group exhibited highly significant improvements in pain (VAS) (p=0.000), suggesting effective pain management. Disability (ODI) significantly decreased (p=0.003), indicating enhanced physical functioning. Physical health (PHY) also improved (p=0.014), showing the benefits of integrating Ayurveda with Yoga. Additionally, depression levels significantly reduced (BDI) (p=0.006), indicating strong mental health benefits. However, no significant changes were observed in psychological health (PSYCH), social health (SOC), and environmental health (ENV), suggesting these areas may need more targeted interventions beyond Yoga and Ayurveda (Table 4).

**Table 4:** Within-group and between-group analyses for variables.

	Mean ± SD			Within group				Between Group		
				Baseline vs 1 Weeks		Baseline vs 3 months				
	Baseline	1 Week	3 Months	% change	P value	% change	P value	F	P value	Partial Eta Squared
VAS										
YA	5.89 ± 1.45	4.17 ± 1.38	4.72 ± 1.57	29.20	0.000***	19.86	0.004**	1.731	0.197	0.047
YO	5.58 ± 1.50	3.74 ± 1.19	3.95 ± 1.35	32.97	0.000***	29.21	0.000***			
ODI										
YA	35.33 ± 14.11	24.78 ± 15.81	28.33 ± 12.64	28.86	0.003**	19.81	0.004**	3.888	0.057	0.100
YO	28.95 ± 11.73	19.05 ± 11.20	20.11 ± 8.42	34.20	0.001**	30.53	0.005**			
PHY										
YA	57.39 ± 11.08	64.44 ± 8.54	59.83 ± 12.90	12.28	0.014*	4.25	0.362	1.031	0.317	0.029
YO	59.00 ± 14.26	64.95 ± 9.78	66.53 ± 10.79	10.84	0.066	12.76	0.033*			
PSYCH										
YA	63.67 ± 13.37	68.50 ± 12.00	66.44 ± 9.56	7.59	0.165	4.35	0.075	0.819	0.372	0.023
YO	65.58 ± 15.08	71.74 ± 12.48	70.53 ± 10.51	9.39	0.085	7.54	0.036*			
SOC										
YA	66.06 ± 13.11	71.56 ± 8.08	73.28 ± 4.73	8.32	0.161	10.93	0.030*	0.702	0.408	0.020
YO	72.42 ± 7.24	69.63 ± 16.12	73.68 ± 4.50	3.85	0.516	1.73	0.535			
ENV										
YA	70.56 ± 9.12	74.00 ± 4.24	73.39 ± 6.89	4.88	0.060	4.01	0.027*	1.113	0.299	0.031
YO	73.74 ± 9.93	76.11 ± 9.85	75.42 ± 6.73	3.21	0.276	2.28	0.252			
BDI										
YA	9.00 ± 3.25	6.61 ± 3.20	8.50 ± 2.88	26.26	0.006**	5.56	0.48	0.227	0.637	0.006
YO	10.00 ± 4.44	7.21 ± 4.10	8.26 ± 3.09	27.9	0.014*	17.4	0.045*			

(Abbreviations: ***P < .001, **P < .01, *P < .05 Repeated measures ANOVA, YA=Yoga and Ayurveda (n = 18), Y= Yoga-only.

(n = 19), VAS=Visual analogue scale, ODI= Oswestry Low Back Pain Disability Questionnaire, PHY= Physical health, PSYCH = Psychological health, SOC=Social health, ENV = Environment, area domains of WHOQOL-BREF, BDI= Beck's Depression Inventory).

### 3.2 Yoga-only

After one week and three months, Yoga-only group showed highly significant improvements in pain levels (VAS) (p=0.000) and significantly reduced disability (ODI) (p=0.001). Depression levels also decreased significantly (BDI, p=0.014). However, there were no significant improvements in physical health (PHY) (p=0.066), psychological health (PSYCH) (p=0.085), social health (SOC) (p=0.516), or environmental health (ENV) (p=0.276). These findings suggest that while Yoga alone effectively manages pain, disability, and depression, it may not significantly impact other health areas (Table 4).

### 3.3 Between groups

The repeated measures between-group analysis, conducted via ANOVA, showed that there was no significant interaction effect between the groups and time concerning pain, disability, depression, and quality of life (refer to Table 4). Despite patients in the Yoga and Ayurveda group initially experiencing more problems, they subjectively reported greater benefits. Although these trends lacked statistical significance, they suggest potential advantages in the Yoga and Ayurveda groups. It is imperative to acknowledge that conducting further research with larger sample sizes and longer intervention durations may be necessary to provide more conclusive results.

## 4 Discussion

In the current study, individuals with persistent low back pain showed comparable improvements in depression, pain, disability, and physical performance, after participating in either the residential yoga program (IAYT) or a 1-week IAYT with *Kati Basti.* This study is likely the first to evaluate the effects of IAYT and IAYT combined with *Kati Basti*, an Ayurvedic therapy, impact on persistent low back pain.

In a prior study of a week-long yoga program, baseline PSS scores inversely correlated with WHOQOL measures. Yoga notably enhanced quality of life across all WHOQOL domains compared to the control group [23]. CLBP patients showed better results with a seven-day intensive yoga program than with physical exercise, showing decreased pain and improved spine flexibility [11,23]. A study on nurses found that practicing yoga improves the social, psychological, and physical aspects of quality of life [27]. Yoga can help CLBP patients function emotionally, reduce pain catastrophizing, increase pain acceptance, improve joint flexibility and muscle strength, and lessen pain-related disability [28]. It has been demonstrated that Yoga helps lumbar spondylitis patients feel less pain and disabled [29]. More effective pain treatment for low back pain is achieved when standard physiotherapy is combined with yoga therapy [30]. Similar results were obtained in this study, confirming the findings of earlier research.

A study suggests that combining Agnikarma and *Kati Basti* with *Ksheerbala Taila* can enhance treatment efficacy for *Gridhrasi* (Sciatica) patients [31]. *Kati Basti* with *Sahacharandi Taila*, and *Mahanarayan* *Taila* were similarly effective in reducing pain and improving walking distance [32]. Additionally, patients with CLBP reported significant improvements after undergoing a combination of *Panchatikta* *Kshira Basti*, *Kati Basti*, and Trayodashang Guggulu treatment [18].

Yoga treatment may have a dual effect on chronic low back pain (CLBP) by promoting parasympathetic function and reducing hypothalamic-pituitary-adrenal (HPA) activity [33]. The yoga intervention involves lifestyle factors such as stress management [34], and diet, along with practices like asana, pranayama, relaxation techniques, meditation, yogic counseling. Individuals with chronic pain can benefit from a combination of asana, pranayama, and meditation with these lifestyle modification factors to alleviate anxiety, depression, and enhance quality of life. Mindfulness and relaxation are crucial in reducing the sympathetic nervous system (SNS) and HPA activity [35]. Yoga has been shown to improve physical performance, reduce depression, increase blood serotonin levels, increase BDNF levels, and regulate pain perception pathways [36]. Yoga therapy maintained the amount of TNF while lowering serum cortisol [37]. Yoga therapy lowers TNF-alpha, a key inflammatory factor in sciatica and disc-related low back pain, offering potential relief for lumbar radicular pain [38]. Yoga therapy enhances the flexibility and strength of muscles as well as respiratory and cardiovascular health, contributing to overall musculoskeletal well-being [39]. Yoga poses to aid in relaxing muscles and joints, potentially improving musculoskeletal health [33,40]. Yoga appeared to be more beneficial than exercise in alleviating CLBP, possibly due to factors beyond its physical aspects. Key elements such as posture maintenance, breath control, mindfulness, and relaxation [41].

The strengths of this study include its focus on both Yogic and Ayurveda sciences. No previous study has combined Integrative Approach to Yoga Therapy (IAYT) with Ayurveda therapy of *Kati Basti* for chronic low back pain. This study is the first to assess the combined effect of IAYT and *Kati Basti* versus IAYT alone. Although the intervention was short-term, the 3-month follow-up showed good acceptability and adherence to therapy. The significant within-group results enhance the program's acceptability in today's fast-paced society.

This study is designed to evaluate the combining IAYT and *Kati Basti* for long-term low back pain, recognizing the importance of understanding their effects over time. The smaller sample size and the one-week duration posed a constraint. Sharing the same arrangement between groups made it difficult to rule out communication biases.

The limitation of this study was the somewhat small sample size. As a result, applying the study's findings to a larger population would not be unquestionably conclusive. Since both groups were using the same setup, it was impossible to completely rule out the potential for some interaction and ideasharing. Upon discharge, patients were asked to continue the practices daily; however, *Kati Basti* cannot be replicated in a home setup.

Future research should be conducted using a larger sample size. Utilizing more objective measurements like spine MRIs, EMG tests, and X-rays before and after, as well as tools for assessing psychological variables like stress and anxiety, and looking for synergistic outcomes by combining these therapies with other complementary and alternative medicine (CAM) therapies like physiotherapy, naturopathy, and acupuncture. The results indicate that people with chronic low back pain may benefit from a one-week residential Yoga program in terms of their overall well-being. However, more study is required to determine the long-term impacts and variations in outcomes between various yoga programs.

## 5 Conclusions

For individuals experiencing persistent lower back pain, a one-week residential yoga program demonstrated significant improvements in pain, disability, physical performance, and depression, either independently or when combined with *Kati Basti* treatment. Although patients in the Yoga and Ayurveda group initially experienced more challenges, they reported greater subjective benefits. It is essential to acknowledge that substantiating these findings necessitates further research employing large sample sizes. Enhanced validation of the effectiveness of residential yoga programs in managing persistent low back pain will emerge through the promotion of more comprehensive investigations.

## Funding sources

The research study did not receive any grant from funding agencies.

## Conflict of interest

Authors have no conflict of interest.

## Declaration of generative AI in scientific writing

None.

## Author contributions

**MB:** Conceptualization, Writing original draft, & Study design, **SSP:** Conceptualization, Investigation, Visualization, Data curation, Supervision, Project administration, & Resources. **SSY:** Writing-original draft, Writing-review & editing, Formal analysis, & Software. **SS:** Writing-original draft, Writing-review & editing, **RT**: Writing-review & editing.

## Data availability

Data can be obtained from the author on request.

## References

[1] Dionne C.E., Dunn K.M., Croft P.R., Nachemson A.L., Buchbinder R., Walker B.F. (2008). A consensus approach toward the standardization of back pain definitions for use in prevalence studies. Spine.

[2] Besen E., Young A.E., Shaw W.S. (2015). Returning to work following low back pain: towards a model of individual psychosocial factors. J Occup Rehabil.

[3] Van Tulder M.W., Assendelft W.J.J., Koes B.W., Bouter L.M. (1997). Spinal radiographic findings and nonspecific low back pain. A systematic review of observational studies. Spine.

[4] Paolucci T., Attanasi C., Cecchini W., Marazzi A., Capobianco S.V., Santilli V. (2019). Chronic low back pain and postural rehabilitation exercise: a literature review. J Pain Res.

[5] Clark S., Horton R. (2018). Low back pain: a major global challenge. Lancet.

[6] Crombez G., Vlaeyen J.W.S., Heuts P.H.T.G., Lysens R. (1999). Pain-related fear is more disabling than pain itself: evidence on the role of pain-related fear in chronic back pain disability. Pain.

[7] Ganesan S., Acharya A.S., Chauhan R., Acharya S. (2017). Prevalence and risk factors for low back pain in 1,355 young adults: a cross-sectional study. Asian Spine J.

[8] Allegri M., Montella S., Salici F., Valente A., Marchesini M., Compagnone C. (2016). Mechanisms of low back pain: a guide for diagnosis and therapy. F1000Research.

[9] Doualla M., Aminde J., Aminde L.N., Lekpa F.K., Kwedi F.M., Yenshu E.V. (2019). Factors influencing disability in patients with chronic low back pain attending a tertiary hospital in sub-Saharan Africa. BMC Muscoskel Disord.

[10] Delitto A., George S.Z., Van Dillen L.R., Whitman J.M., Sowa G., Shekelle P. (2012). Low back pain. J Orthop Sports Phys Ther.

[11] Tekur P., Nagarathna R., Chametcha S., Hankey A., Nagendra H.R. (2012). A comprehensive yoga programs improves pain, anxiety and depression in chronic low back pain patients more than exercise: an RCT. Compl Ther Med.

[12] Ward L., Stebbings S., Cherkin D., Baxter G.D. (2013). Yoga for functional ability, pain and psychosocial outcomes in musculoskeletal conditions: a systematic review and meta-analysis. Muscoskel Care.

[13] Saper R.B., Sherman K.J., Delitto A., Herman P.M., Stevans J., Paris R. (2014). Yoga vs. physical therapy vs. education for chronic low back pain in predominantly minority populations: study protocol for a randomized controlled trial. Trials.

[14] Cox H., Tilbrook H., Aplin J., Chuang L.H., Hewitt C., Jayakody S. (2010). A pragmatic multi-centred randomised controlled trial of yoga for chronic low back pain: trial protocol. Compl Ther Clin Pract.

[15] Upadhyay J., S N.N., Shetty S., Saoji A.A., Yadav S.S. (2023). Effects of Nadishodhana and Bhramari Pranayama on heart rate variability, auditory reaction time, and blood pressure: a randomized clinical trial in hypertensive patients. J Ayurveda Integr Med.

[16] Kj S., Nk M., Pg A. (2023). Ayurveda, yoga, and acupuncture therapies in alleviating the symptom score among patients with spinal cord injury – a systematic review. J Ayurveda Integr Med.

[17] Erhss Ediriweera, Hdp Gunathilka, Weerasinghe K.D.C.M., Otmrksb Kalawana (2013). Efficacy of traditional treatment regimen on Kati Shoola with special reference to lumbar spondylolisthesis. Ayu.

[18] Kaalia N., Bhatted S.K., Acharya S. (2022). Effect of *Panchatikta Ksheera basti* with *Kati basti in Katishoola* w. s. r lumbar disc degeneration – a clinical study. Indian J Heal Sci Biomed Res.

[19] Kulkarni M., Shereka D., Deshpande P. (2017). “Pharmacodynamic understanding of katibasti” - a contemporary approach. Int J Adv Res.

[20] Bhusal N., Mangal G. (2021).

[21] Kaushal M, Vagul N, Dwivedi R, Singh AK. Management of Low Back Pain : Through Ayurveda-A Review n.d.;2:17–18. 10.5281/zenodo.3551305.

[22] Rao V.N., Shankar T., Dixit S.K., Ray A.B. (1996). Standardisation of ksheerabala taila. Ancient Sci Life.

[23] Tekur P., Chametcha S., Hongasandra R.N., Raghuram N. (2010). Effect of yoga on quality of life of CLBP patients: a randomized control study. Int J Yoga.

[24] Patil N.J., Nagarathna R., Tekur P., Patil D.N., Nagendra H.R., Subramanya P. (2015). Designing, validation, and feasibility of integrated yoga therapy module for chronic low back pain. Int J Yoga.

[25] Fairbank J.C.T., Pynsent P.B. (2000). The Oswestry disability Index. Spine.

[26] Richter P., Werner J., Heerlein A., Kraus A., Sauer H. (1998). On the validity of the Beck depression inventory. A review. Psychopathology.

[27] Patil N., Nagaratna R., Tekur P., Manohar P., Bhargav H., Patil D. (2018). A randomized trial comparing effect of yoga and exercises on quality of life in among nursing population with chronic low back pain. Int J Yoga.

[28] Holtzman S., Beggs R.T. (2013). Yoga for chronic low back pain: a meta-analysis of randomized controlled trials. Pain Res Manag J Can Pain Soc.

[29] Manik R., Mahapatra A., Gartia R., Bansal S., Patnaik A. (2017). Effect of selected yogic practices on pain and disability in patients with lumbar spondylitis. Int J Yoga.

[30] M R.Y.B., J E., J R. (2016). Efficacy of yoga therapy (IAYT) on pain in patients undergoing conventional physiotherapy for chronic low back ache. J Pharmaceut Sci Innovat.

[31] Bali Y., Ebnezar J., Venkatesh B., Vijayasarathi R. (2010). Efficacy of Agnikarma over the padakanistakam (little toe) and Katibasti in Gridhrasi: a comparative study. Int J Ayurveda Res.

[32] Mangal G., Garg G., Shyam Sr (2013). OA03.18. “A comparative study of kati basti with sahacharadi taila and maha narayana taila in the management of gridhrasi (Sciatica).”. Ancient Sci Life.

[33] Ross A., Thomas S. (2010). The health benefits of yoga and exercise: a review of comparison studies. J Alternative Compl Med.

[34] Yadav S.S., Saoji A.A., Somanadhapai S., Yadav N.L., Upadhyay J., Rishi N.N., Thapa R. (2024). Effect of Yoga-based breathing practices on depression, anxiety, stress, and fear of COVID-19 positive hospitalized patients: A randomized controlled trial. J. Ayurveda Integr. Med..

[35] Vallath N. (2010). Perspectives on yoga inputs in the management of chronic pain. Indian J Palliat Care.

[36] Lee M., Moon W., Kim J. (2014). Effect of yoga on pain, brain-derived neurotrophic factor, and serotonin in premenopausal women with chronic low back pain. Evidence-Based Complement Altern Med.

[37] Cho H.K., Moon W., Kim J. (2015). Effects of yoga on stress and inflammatory factors in patients with chronic low back pain: a non-randomized controlled study. Eur J Integr Med.

[38] Wang H., Schiltenwolf M., Buchner M. (2008). The role of TNF-α in patients with chronic low back pain - a prospective comparative longitudinal study. Clin J Pain.

[39] Woodyard C. (2011). Exploring the therapeutic effects of yoga and its ability to increase quality of life. Int J Yoga.

[40] Ross A., Thomas S. (2010). The health benefits of yoga and exercise: a review of comparison studies. J Alternative Compl Med.

[41] Govindaraj R., Karmani S., Varambally S., Gangadhar B.N. (2016). Yoga and physical exercise - a review and comparison. Int Rev Psychiatr.

